# The prevalence of sexual violence against African women: a systematic review and meta-analysis

**DOI:** 10.4314/ahs.v23i3.15

**Published:** 2023-09

**Authors:** Qu Xianguo, Cao Hui, Shen Xin, Feng Jing, Wang Zijian, Niu Zhenyu, Gan Yong

**Affiliations:** 1 Zhejiang University School of Medicine, Affiliated Hangzhou First People's Hospital; 2 Beijing Vocational College of Labour and Social Security, Department of Labor Economics and Management; 3 Huazhong University of Science and Technology, Department of Social Medicine and Health Management; 4 Guangxi University of Science and Technology, School of Arts and Communication; 5 Shihezi University, Department of Public Health and Preventive Medicine

**Keywords:** Sexual violence, African women, Meta-analysis

## Abstract

**Background:**

High rates of sexual violence ratios in low-income countries are recognized as a global public health problem. The incidence of violence against African women has been increasing. However, no study has systematically summarized the global prevalence of sexual violence against African woman.

**Methods:**

We conducted a comprehensive search of PubMed, Embase and Web of Science, databases from their inception through January 2021 for pertinent studies on reporting the prevalence of sexual violence against African women. We included observational studies. The prevalence rate was estimated using a random-effects meta-analysis. The heterogeneity was evaluated using I2 statistic. Differences by study level characteristics were estimated through subgroup analysis and meta-regression.

**Results:**

A total of 9 cross-sectional studies were included (a total of 9,030 participants). The pooled sexual violence rate was 0.33 (95% CI = 0.23–0.42). Subgroup analyses found that there was a higher rates of sexual violence against pregnant woman in east Africa (0.41, 95% CI = 0.24–0.58), pregnant (0.42, 95% CI = 0.05–0.80), and interview (0.40, 95% CI = 0.01–0.78). The analysis found that the major sexual violence types were the physical violence (0.19, 95% CI = 0.07–0.31), psychological violence (0.36, 95% CI = 0.11–0.61), sexual assault (0.25, 95% CI = 0.02–0.47).

**Conclusions:**

Nearly one out of every three (33%) African woman around the world has been a victim of sexual violence in their life. This current study investigated the status and characteristics of sexual violence against women, which could provide an important reference for the African health care provider. Assessing this problem against African women helps government officials, policy makers, program designers and non-governmental organizations to design prevention and controlling strategies.

## Background

The scale of sexual violence is staggering in developing country, especially in Africa. It includes, but is not limited to, rape within marriage or dating relationships; rape by strangers or acquaintances; unwanted sexual advances or sexual harassment, including at work and at school; systematic rape, sexual slavery and other forms of violence^1^, which are particularly common in armed conflicts; the sexual assault of people with physical and mental disabilities; the sexual abuse of children; and ‘customary’ forms of sexual violence such as forced marriage or cohabitation and wife inheritance^2^.

The incidence of violence against African women has been increasing despite efforts by law enforcement orders^3^. Different characteristics may associate with sexual violence against pregnant women 4. Identifying these characteristics in a given society is the critical step to minimize the frequency of sexual violence and adverse outcome on maternal and child health. Some characteristics are well-known factor for sexual violence. In the broadest sense, violence against African women is any violation of a woman's personhood, mental or physical integrity or freedom of movement. Violence against African women is considered as an obstacle to the achievement of the objectives of equality, development and peace^4^. Moreover, the act violates and impairs African women's rights and fundamental freedoms. The low social and economic status of African women can be both a cause and a consequence of violence against women^6^. Physical, sexual and psychological violence occurring in the family, including battering, sexual assault of female children, dowry-related violence, marital rape, femalgenital mutilation and other traditional practices harmful to women, limit the ability to make choices on African women's lives^7^.

Although any woman can be a victim of rape at any time, the chance of suffering sexual violence appears to be greater against low-income countries women, including young girls and adolescents, particularly if they live alone, with only one parent or with a stepfather 8. The perpetrators are often their partners, a member of the family or another person with authority over them 8. Moreover, among adolescents, their first experience of sexual intercourse is frequently under force or coercion, and this is more common when it happens very early in life 1.

High rates of sexual violence ratios in low-income countries are recognized as a global public health problem. However, the problem is neglected in low-income countries even though all proclaim that without addressing violence against women it is difficult to achieve expected growth and development targets 10. A large proportion of women believe that it is acceptable for a man to beat his wife. This perception perpetuates the violence against women and needs to be changed 11.

Sexual violence is far more prevalent in daily life in African societies than is usually suspected. However, no study has systematically measured the global prevalence of sexual violence against African woman. This current study investigated the status and characteristics of sexual violence against women, which could provide an important reference for the African health care provider. Assessing this problem against African women helps government officials, policy makers, program designers and non-governmental organizations to design prevention and controlling strategies^12^.

## Methods

### Search strategy and selection criteria

A systematic review and meta-analysis following the MOOSE guidelines was performed to assess the status and factors related to sexual violence against women. 13, 14 The search strategy is based on the PRISMA 2020 guideline. We conducted a comprehensive search of PubMed, Embase and Web of Science, databases from inception through January 2021 for pertinent studies on sexual violence against African women. The search terms were “sexual violence or sex violence or sexual assault or African female or African woman or African women or African girls.” Only articles published in English were considered. Moreover, we manually scrutinized the reference lists of the retrieved articles for additional relevant articles.

All of the studies retrieved by the comprehensive search were screened by title or abstract and then by a full-text assessment. We began selecting articles by screening the titles and abstracts of the articles retrieved from the database search. When relevance could not be determined by screening titles and abstracts, the full text was reviewed. Then, the full texts of all the articles assessed as possibly relevant were reviewed.

Two researcher (X.S. and L.L.) chose potentially relevant articles based on the titles or abstracts, and two other researchers (H.C. and J. F.) reviewed those articles to build the final dataset based on the following inclusion criteria: (1) observational study design (cross-sectional, case-control and cohort studies), (2) sample defined as women aged 14 years or older, (3) the concept of sexual violence meets the above-mentioned definition of sexual violence and (4) the article reported the rate of sexual violence against women or provided sufficient information for it to be calculated. We excluded reviews, essays, letters, and commentaries. When multiple articles reporting the same study sample were identified, the article with the most complete information on results or that reported on the largest number of cases was chosen for the dataset.

### Data extraction

Two researchers (X.S. and L.L.) separately performed the data extraction, compared their results, and resolved inconsistencies by reaching consensus through discussion. We used a predefined and standardized data extraction form developed specifically for this study to collect information from the dataset. These data were author names, years of publication, country, sample sizes, and, regarding physical violence, psychological violence and sexual assault as the characteristics identified by the studies as associated with sexual violence against women. In cases where the information was not in articles, it was requested from the articles' corresponding authors.

### Quality assessment

To assess the quality of the studies reported by the articles in the dataset, we used an 11-item index recommended by the Agency for Healthcare Research and Quality.^15^ Three items assessed the quality of the studies' sample selection methodology (e.g., inclusion/exclusion criteria), five items assessed the quality of the variables (e.g., data source, reliability/validity assessment statistics), and three items assessed the quality of the analytical methods (e.g., management of missing data, extent of confounding variables). The response options were yes, no, or unsure. The scoring system assigned one point to articles that indicated the study included the item (yes) and zero points when information was missing (no) or we were unable to determine whether it had been considered (unsure). The scores ranged from zero to 11 points, with higher scores indicating higher quality. Supplement Table 1 reports the distribution of scores. Two of the researchers reviewed the quality ratings of the articles' studies, and inter-rater reliability on titles, abstracts, and full-text screenings was determined using Cohen's ϰ.

**Supplementary Table 1:** Quality assessment of cross-sectional studies*

Author	Year	1) Define the source of information (survey, record review)	2) List inclusion and exclusion criteria for exposed and unexposed subjects (cases and controls) or refer to previous publications	3) Indicate time period used for identifying patients	4) Indicate whether or not subjects were consecutive if not population-based	5) Indicate if evaluators of subjective components of study were masked to other aspects of the status of the participants	6) Describe any assessments undertaken for quality assurance purposes	7) Explain any patient exclusions from analysis	8) Describe how confounding was assessed and/or controlled.	9) If applicable explain how missing data were handled in the analysis	10) Summarize patient response rates and completeness of data collection	11) Clarify what follow-up, if any, was expected and the percentage of patients for which incomplete data or follow-up was obtained	Total quality score
Iryna et al	2007	1	1	1	1	1	0	1	1	0	1	0	8
Parveen et al	2012	1	1	1	1	1	0	0	1	0	1	1	8
Margaret et al	2014	1	1	1	0	1	0	1	1	1	1	1	9
Verelst et al	2014	1	0	1	1	1	1	0	1	1	1	1	9
Stephen et al	2015	1	1	1	0	1	0	0	0	1	1	1	7
Jennifer et al	2015	1	1	1	0	1	1	1	1	0	1	0	8
Akashi et al	2016	1	0	1	1	1	0	0	0	0	1	1	6
Elfalet et al	2018	1	1	1	1	1	0	0	1	1	1	1	9
Bikila et al	2019	1	1	1	0	1	1	0	1	1	1	1	9

* The study quality was assessed according to the 11 items recommended by the Agency for Healthcare Research and Quality (AHRQ) for cross-sectional studies. 1 point if the item was contemplated in the study, 0 point if the item was not, and unable to determine. 1 = “Yes”, 0 = “No”, “Unable to determine”, or “Not applicable”

### Data analysis

Sexual violence rate against African woman was calculated in the meta-analysis using a random effects model. The extent of statistical heterogeneity across the articles was estimated by I^2^, and the values at 25 percent, 50 percent, and 75 percent were the cut-off points of low, moderate, and high heterogeneity, respectively. 16

Sensitivity analyses were used to investigate the sources of heterogeneity. Variations in the sexual violence rates were tested by publication year, study location, only pregnant/all women, participants age, survey method, and studies quality. Study quality and group differences were tested to investigate heterogeneity across groups. Group analyses by publication year, study location, developmental level, survey method, studies quality was performed to examine the influences of associated factors. All the group differences were tested in meta-regression analyses. 17 Publication bias was assessed using the funnel plot 18. All statistical analyses were performed in STATA 12.0. Except as otherwise specified, all tests of significance were two-tailed and the cut-off value of statistical significance was P<0.05.

## Results

### Study selection

**Figure 1:**
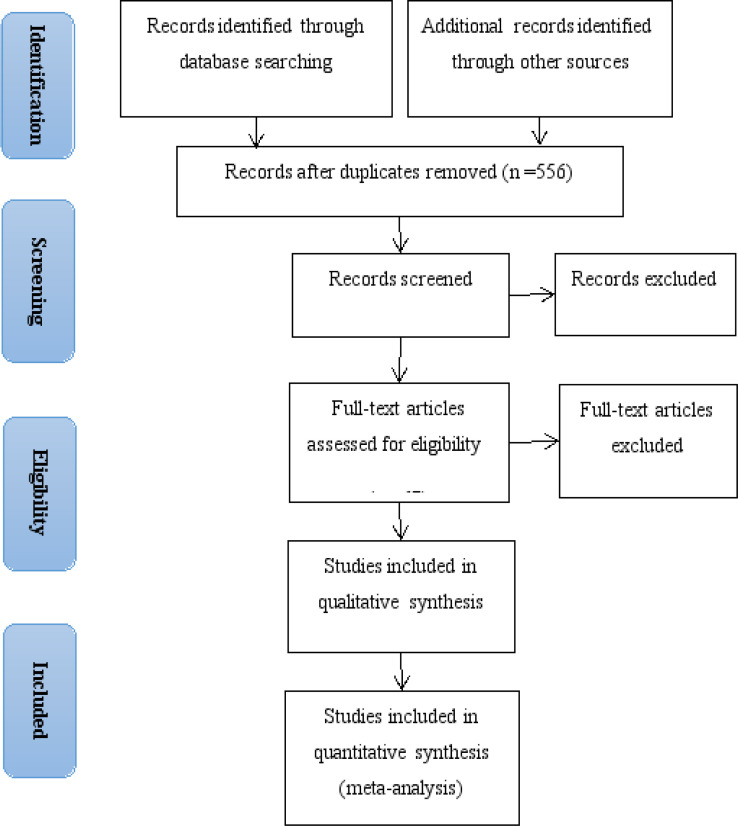
Flow chart of identification of relevant observational studies

Figure 1 illustrates the study selection, identification, and inclusion process using the Preferred Reporting Items for Systematic Reviews and Meta-Analyses flow chart. First, 1,131 articles were retrieved from the PubMed, Embase and Web of Science. After the initial screening of titles and abstracts, 67 articles remained for full-text assessment. After the detailed full-text evaluation, 19 studies comprised the analytical sample. Of them, three articles' data reports were insufficient and two articles reported on the same study, resulting in 9 articles published between 2006 and 2019 in the quantitative synthesis. 19-27

### Article and study characteristics

The main characteristics of the 10 articles in the dataset are shown in Table 1. Four were conducted in East Africa, two were conducted in Central Africa and three were conducted in West Africa. All of the articles reported results on African women. The sample sizes ranged from 251 to 3,422 (median = 600, interquartile range =413– 1305), and the total number of cases was 9,030. Observer agreement (ϰ) was 0.93, indicating excellent agreement between ratters for article inclusion determination. The sample sizes ranged from 6 to 9 (median = 8.11). The quality assessment scores were nine or higher in 5 articles on the 0–11-point scale (Supplementary Table 1).

**Table 1:** Characteristics of studies included in the meta-analysis

Author	Year	Country	Age at baseline, years	Survey method	No. of participants
Iryna et al	2007	Uganda	15-24	interview	3422
Parveen et al	2012	Cameroon	≥14	questionnaire	600
Margaret et al	2014	Nigeria	≥14	questionnaire	413
Verelst et al	2014	Congo	11-23	questionnaire	1305
Stephen et al	2015	Uganda	≥15	questionnaire	1307
Jennifer et al	2015	Gambia	≥16	questionnaire	251
Akashi et al	2016	Rwanda	≥14	questionnaire	921
Elfalet et al	2018	Ethiopia	18-49	questionnaire	450
Bikila et al	2019	Ethiopia	15-45	interview	361

### Prevalence of sexual violence against women

**Figure 2:**
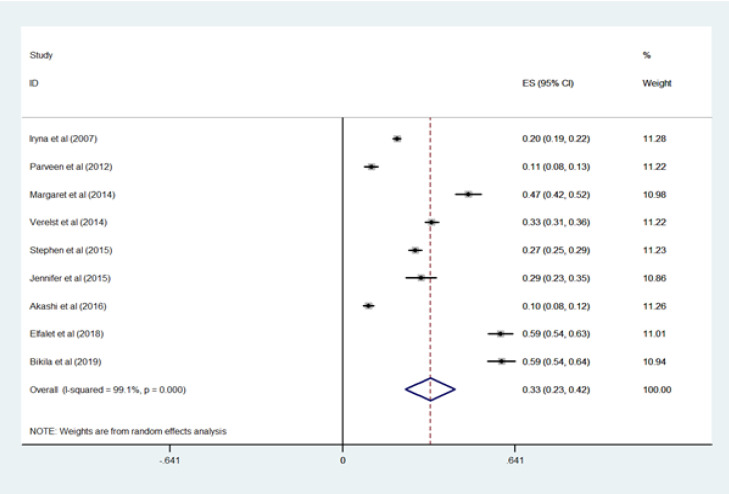
Pooled random effects prevalence rate and 95% Confidence Interval

The pooled sexual violence rate was 0.33 (95% CI = 0.23–0.42), indicating that about 33% of the women had suffered from the sexual violence. The heterogeneity was I^2^ =99.1% (Figure 2).

### Subgroup analysis

There were no significant differences in rates against publication year and studies quality. Groups comparisons found that sexual violence rate data obtained from east Africa (0.41, 95% CI = 0.24–0.58) had much higher rates than those whose data were obtained from central Africa (0.21, 95% CI = -0.02–0.44) and west Africa (0.29, 95% CI =0.06–0.52), rate data obtained from only pregnant (0.42, 95% CI = 0.05-0.80) had much higher rates than the data were obtained from all women (0.28, 95% CI = 0.30–0.36), rate data obtained from only young women(≤ 24) (0.34, 95% CI = 0.20–0.48) had much higher rates than the data were obtained from all women (0.23, 95% CI = 0.14–0.39), and interview (0.40, 95% CI = 0.01–0.78) had much higher rates than those whose data were obtained by questionnaire (0.31, 95% CI = 0.19–0.42) (Table 2). Variability was studied after adding study location, only pregnant or all women, participants age and survey method, and the results of meta-regression showed that these factors could explain 76.71% variability.^28^

**Table 2:** Subgroups analyses of prevalence rate of sexual violence among African women

	No. of reports	Prevalen rate (%)	ce Lower Limit (LL)	Upper Limit (UL)	*I*^*2*^(%)	*P* for heterogeneity	*P* value* between groups
**Primary analysis**	9	0.33	0.23	0.42	99.10%	<0.001	0.019
**Subgroup analyses**							
**Publication year**							
-2015	4	0.28	0.16	0.39	98.90%	<0.001	0.628
2015-1019	5	0.37	0.18	0.55	99.40%	<0.001	
**Study location**							
East Africa	4	0.41	0.24	0.58	99.30%	<0.001	0.023
Central Africa	2	0.21	-0.02	0.44	99.50%	<0.001	
West Africa	3	0.29	0.06	0.52	98.90%	<0.001	
**Only pregnant/All women**							
Only pregnant	6	0.42	0.05	0.80	98.20%	<0.001	0.046
All women	3	0.28	0.20	0.36	99.70%	<0.001	
**Participants age**							
Only young women (≤ 24)	2	0.23	0.14	0.39	98.70%	<0.001	0.098
All women	7	0.34	0.20	0.48	99.30%	<0.001	
**Survey method**							
Questionnaire	7	0.31	0.19	0.42	99.10%	<0.001	0.036
Interview	2	0.40	0.01	0.78	99.50%	<0.001	
**Studies quality**							
<9	5	0.19	0.12	0.26	97.80%	<0.001	0.149
>9	4	0.49	0.35	0.63	97.90%	<0.001	

* *P* values for meta-regression.

### Types associated with sexual violence against women

Four articles reported the rates of associated sexual violence types. (19, 25-27) The analysis found that the major characteristics were the physical violence (0.19, 95% CI = 0.07–0.31), psychological violence (0.36, 95% CI = 0.11–0.61), sexual assault (0.25, 95% CI = 0.02–0.47). (Table 3).

**Table 3:** Meta-analysis of types of sexual violence against African women

Type	No. of reports	Prevalence rate (%)	Lower Limit (LL)	Upper Limit (UL)	*I*^*2*^(%)	*P* for heterogeneity	*P* value* between groups
Physical violence	3	0.19	0.07	0.31	98.90%	<0.001	0.01
Psychological violence	3	0.36	0.11	0.61	99.20%	<0.001	0.05
Sexual assault	2	0.25	0.02	0.47	98.70%	<0.001	0.03

* *P* values for meta-regression.

### Sensitivity analyses

Sensitivity analyses were performed to explore potential sources of between-study heterogeneity. The pooled rate was not materially changed in the leave-one-out analyses by omitting one study in turn. The pooled sexual violence rate was not materially changed in the leave-one-out analyses by omitting one study in turn from 0.26 (95%CI: 0.22-0.29, I^2^ = 92.1%) to 0.35 (95%CI: 0.33-0.37, I^2^ = 99.8%).

### Publication bias

**Figure 3:**
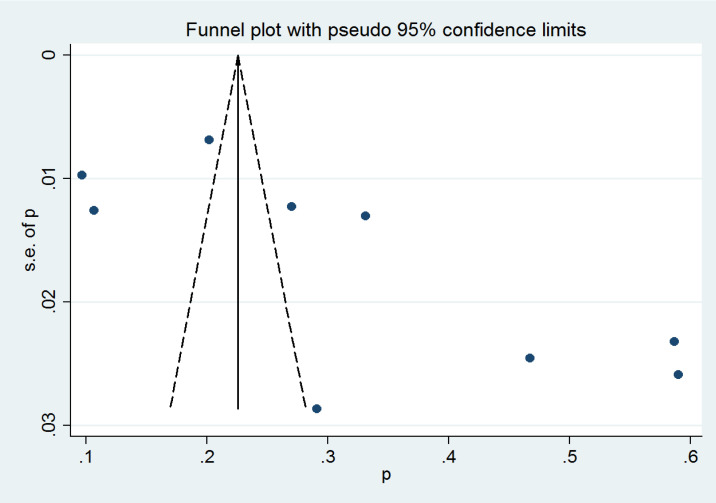
Funnel plot of the prevalence of sexual violence

A funnel plot was generated (Figure 3) and visual inspection of it revealed the asymmetry. It is worth noting that publication bias usually refers to the tendency of authors or publishers of papers to select positive results for publication when publishing papers. Although the primary role of the funnel plot here is to check for publication bias, this study is a rate-based meta. The characteristic of this type of studies is precisely that the included studies are not disturbed by whether they are positive or not. Therefore, although the funnel plot of this study is biased to some extent, the results of this study are still not affected by publication bias.

## Discussion

Sexual violence lies behind some of the most intractable reproductive social issues, which undermine a woman's sexual and reproductive autonomy and jeopardize her health. This is the first comprehensive systematic review and meta-analysis on the prevalence of sexual violence against African women. In summary, it is estimated that nearly one out of every three (0.33, 95% CI = 0.23–0.42) women around the world has been a victim of some type of sexual violence in her life.

Some studies measured the sexual violence rate in some African regions, and the results showed that women from east Africa (0.41, 95% CI = 0.24–0.58) had suffered much sexual violence than those from central Africa (0.21, 95% CI = -0.02–0.44) and west Africa (0.29, 95% CI =0.06– 0.52). Our findings provide a more comprehensive reflect of sexual violence rate of different African regions.

Pregnant may suffer from sexual violence (0.42, 95% CI = 0.05–0.80), which could because most of the women may not have sexual desire during pregnancy. Meanwhile, some studies were based on samples of women aged under 24 (0.23, 95% CI = 0.14–0.39), giving a mixture of lifetime experience of violence against women, with short and long periods of exposure to such violence 26. The chances of experiencing sexual violence or imposed sexual intercourse under coercion increases throughout life in parallel with the time of exposure to that risk (0.34, 95% CI = 0.20–0.48). Consequently, the prevalence of lifetime experience of sexual violence should be much higher in women coming to the end of their reproductive years than the average presented in such studies.

Moreover, our study measures any individual acts of physical violence (0.19, 95% CI = 0.07–0.31), psychological violence (0.36, 95% CI = 0.11–0.61) and sexual assault (0.25, 95% CI = 0.02–0.47). Physical and psychological violence may lead victims to re-organize their lives, they may change address, telephone number or leave the place where they live, or they may experience phobias, sexual dysfunction or difficult with returning to their normal routines and jobs 25. Moreover, there are no good estimations of the probability of acquiring human immunodeficiency ciency virus (HIV) during a sexual assault, but there is no doubt that if the aggressor is HIV positive, the forced sexual relation exposes a woman to serious risk of infection 29. The sexual transmission of HIV is well established, as is the higher risk for anal sex. This risk increases greatly when the aggression involves injuries of the genital or anal region, as is often the case during sexual violence 26.

## Limitations

This study is the first one to investigate prevalence rate and mainly characteristics associated with sexual violence against African woman, which is essential to raise awareness against global health-care providers. However, this study has some limitations to consider when interpreting and applying the findings. First, the basic information originates from population-based surveys. Many such studies have been performed around the world to evaluate the prevalence of sexual violence. There is great variability in the prevalence found in different studies. This variation may be attributed to the type of population included in the study, socioeconomic diversity and the definitions of violence. Second, a high heterogeneity was observed in the meta-analysis when the estimates were aggregated. This heterogeneity might relate to differences in survey methods, sample size and cultural backgrounds; however, the heterogeneity can be overestimated when studies with large sample sizes are pooled. 30

## Suggestions

Obstetrician–gynecologists and other women's health care providers should screen all women for a history of sexual assault. Clinicians who evaluate survivors of sexual assault in the acute phase must comply with certain medical and legal requirements.

Clinicians should recognize the short-term and long-term health consequences of sexual assault, such as infection, pregnancy, and mental health conditions and manage them appropriately. Moreover, they should incorporate a trauma-informed care framework when assessing the needs of sexual assault survivors.

## Conclusions

Sexual violence continues to be a major public health problem in low-income countries. Nearly one out of every three (33%) African woman around the world has been a victim of sexual violence in their life. Obstetrician–gynecologists and other women's health care providers play a key role in the evaluation and management of sexual assault survivors and should screen routinely for a history of sexual assault. When sexual violence is identified, individuals should receive appropriate and timely care. The prevalence of sexual violence against African woman need to get the collective attention of the whole society.

## Data Availability

Data may be made available by contacting the corresponding author.
